# The Association Between Childhood Trauma and Meaning in Life: The Mediating Role of Self‐Compassion in Iranian Adults

**DOI:** 10.1002/brb3.71800

**Published:** 2026-09-23

**Authors:** Reza Refaei

**Affiliations:** ^1^ Department of Clinical Psychology, Faculty of Medical Sciences Islamic Azad University Semnan Iran

**Keywords:** childhood trauma, meaning in life, mental health, self‐compassion

## Abstract

**Objective:**

Meaning in life is a fundamental component of mental health that can be influenced by early traumatic experiences. This study aimed to investigate the mediating role of self‐compassion in the relationship between childhood trauma and meaning in life.

**Method:**

This cross‐sectional study utilized a correlational design and structural equation modeling (SEM) approach. The study population included individuals aged 18 to 60 residing in Semnan in 2025, with 562 participants recruited through convenience sampling and voluntarily participating in the research (216 men [38.4%], 346 women [61.6%]; M = 40.00, SD = 7.93) The research instruments included the Childhood Trauma Questionnaire (CTQ), the Self‐Compassion Scale (SCS), and the Meaning in Life Questionnaire (MLQ). Data were analyzed using SPSS 26 and Smart‐PLS 4 software.

**Findings:**

The results indicated that childhood trauma had a significant negative effect on self‐compassion (β = −0.462) and meaning in life (β = −0.224). Additionally, self‐compassion showed a significant positive relationship with meaning in life (β = 0.454). The analysis of indirect effects revealed that self‐compassion plays a significant mediating role in the relationship between childhood trauma and meaning in life (β = −0.210, *p* < 0.001).

**Conclusion:**

The findings suggest that self‐compassion can help maintain and enhance meaning in life by reducing the negative consequences of childhood trauma. These results underscore the importance of interventions aimed at fostering self‐compassion in individuals with early traumatic experiences.

## Graphical abstract

Childhood trauma was associated with lower meaning in life both directly and indirectly through reduced self‐compassion. Self‐compassion emerged as a significant protective factor, suggesting that interventions promoting self‐compassion may help mitigate the long‐term psychological impact of early traumatic experiences and enhance meaning in life.

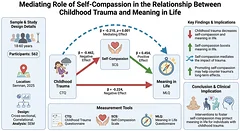

## 1 Introduction

Meaning in life is a subjective experience that makes a person's life meaningful, purposeful, and valuable, encompassing a sense of coherence (understandability and logic of life), having clear goals and direction (purposefulness), and recognizing the inherent value of life (importance) (Martela and Steger 2016). The scientific study of “meaning in life” faces many challenges, as this concept lies at the intersection of conflicting ideas and various definitions. While some view meaning in life primarily as a philosophical or conceptually contested matter, others consider it an important human need associated with psychological well‐being (Schlegel and Hicks 2017; Glaw et al. 2017). In this context, an important distinction exists between “the meaning of life” and “meaning in life”; the former refers to the philosophical question about the ultimate purpose of human existence, while the latter pertains to an individual's subjective and internal experience of feeling purposeful, significant, and coherent in life (King and Hicks 2021). Supporting Information

Frankl, in his logotherapy theory, considered meaning in life to be the core of human existence, stating that humans are inherently in search of meaning and that the “will to meaning” plays a crucial role in mental health (Wolfram 2022). Subsequent research has shown that the experience of meaning is associated with higher levels of well‐being, life satisfaction, and positive affect (Abu‐Raiya et al. 2020; Steger et al. 2006; Russo‐Netzer et al. 2021), and its absence can lead to depression and emotional distress (Baquero‐Tomás et al. 2023; Shek et al. 2022). Given the important role of meaning in life for mental health, researchers have focused on examining factors that may threaten this psychological experience. One such factor is childhood trauma, which encompasses all forms of abuse and neglect during childhood and primarily includes five different types: emotional abuse, emotional neglect, physical abuse, physical neglect, and sexual abuse.

Childhood trauma can impact an individual's emotional and cognitive development and increase the risk of depression, anxiety, and psychological problems in later life (Çiçek and Yıldırım 2026; Refaei and Memari 2025). However, findings indicate that having meaning in life can provide a protective role against the negative consequences of these experiences and assist in the cognitive and emotional reconstruction of past injuries (Çiçek 2025; Liao et al. 2025; Weibel et al. 2017).

According to attachment theory, the capacity for self‐compassion develops within children's early relationships with their caregivers. Secure attachment, by fostering feelings of safety and acceptance, provides a foundation for a kind attitude toward the self; in contrast, childhood maltreatment, through undermining feelings of security and increasing shame and self‐criticism, may interfere with the development of self‐compassion (Zhang et al. 2021). From the perspective of Gilbert's social mentality theory and Neff's conceptualization of self‐compassion, self‐compassion, through reducing self‐criticism and promoting self‐acceptance and emotion regulation, can enhance individuals’ capacity to cope adaptively with difficult experiences and positively reappraise them. This process can strengthen resilience and, ultimately, contribute to the development and maintenance of meaning and purpose in life. Therefore, self‐compassion may function as a psychological mediating mechanism linking childhood maltreatment to meaning in life) Chan et al. 2022; Liao et al. 2025).

Empirical evidence further supports these proposed associations. Evidence has shown that childhood trauma can reduce an individual's capacity for self‐compassion; on the other hand, self‐compassion is associated with an increased experience of meaning in life and can mitigate the negative effects of childhood trauma (O'Dea et al. 2022; Suh and Chong 2021; Tanaka et al. 2011; Wah Ma 2025; Wu et al. 2018; Zhang et al. 2021).

Despite evidence linking childhood trauma, self‐compassion, and meaning in life, limited research has examined how self‐compassion may explain the association between childhood trauma and meaning in life. This study addresses this gap by testing the mediating role of self‐compassion in this association among Iranian adults. Based on previous evidence, it is hypothesized that self‐compassion can help preserve and enhance meaning in life by reducing the negative effects of childhood trauma.

### 1.1 Main Hypothesis

Self‐compassion mediates the relationship between childhood trauma and meaning in life, such that increased self‐compassion can reduce the negative effects of childhood trauma on meaning in life.

### 1.2 Sub‐Hypotheses

1. Childhood trauma is negatively associated with self‐compassion.
2. Childhood trauma is negatively associated with meaning in life.
3. Self‐compassion is positively associated with meaning in life.
4. Self‐compassion mediates the negative association between childhood trauma and meaning in life.

### 1.3 Conceptual Model of the Study

**FIGURE 1:**
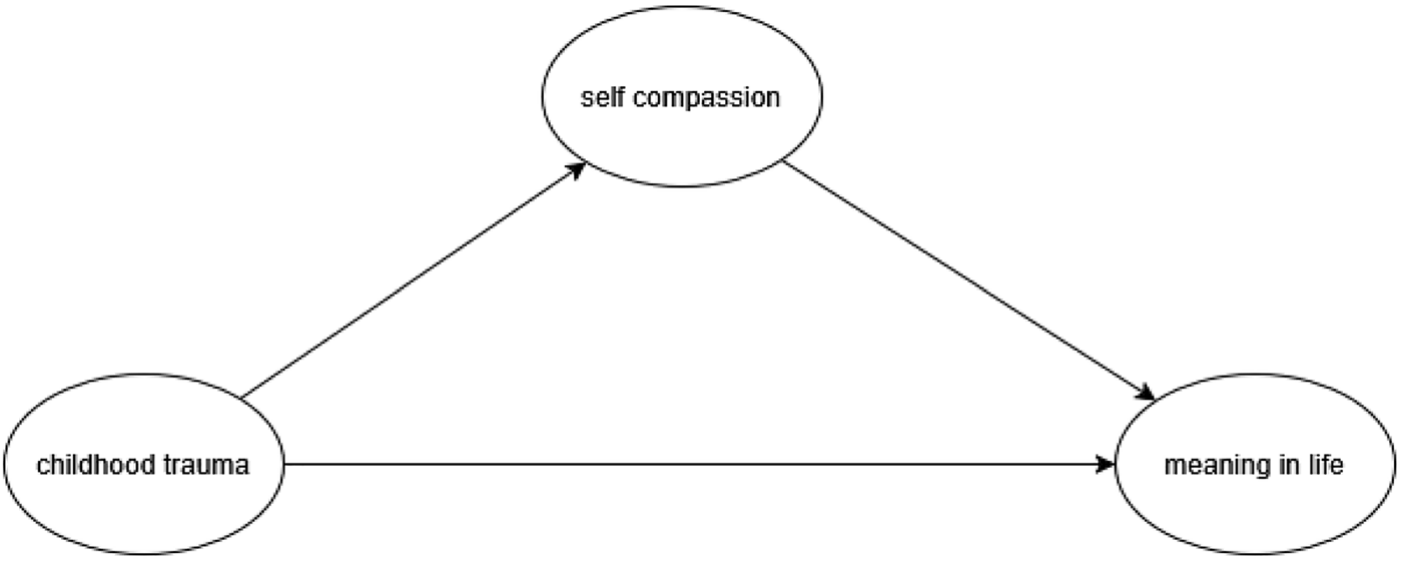
Conceptual model of the study.

This study examines the relationship between childhood trauma, self‐compassion, and meaning in life. It is hypothesized that childhood trauma has a negative effect on meaning in life, and this relationship is partially mediated by self‐compassion, such that self‐compassion can reduce the negative effects of childhood trauma. The proposed mediation model is presented in Figure 1.

## 2 Method

This study was a cross‐sectional design with a correlational approach and utilized structural equation modeling (SEM). The aim of the research was to examine the relationship between childhood trauma, self‐compassion, and meaning in life in individuals aged 18 to 60 in the city of Semnan in 2025.

An a priori power analysis was conducted based on Cohen's statistical power framework using G*Power. Assuming an anticipated effect size of 0.19, a statistical power of 0.90, and a significance level of α = 0.01, the minimum required sample size was calculated to be 562 participants.

### 2.1 Sampling Method

Non‐random convenience sampling was employed, and participants voluntarily participated in the research. This method was chosen for its ease of access to the sample and the possibility of increasing the number of participants within a limited time frame.

Research Implementation Method: Questionnaires were distributed online through relevant Telegram channels. After providing necessary explanations and obtaining informed consent, data were collected through self‐reporting. Incomplete or invalid responses were removed, and the final data were coded and entered into the relevant software for statistical analysis and structural equation modeling.

### 2.2 Inclusion Criteria

Inclusion criteria for the study included being aged 18 to 60, residing in Semnan, and providing informed consent to participate in the research. Participants also needed to have the necessary ability to read and respond to online questionnaires.

### 2.3 Research Exclusion Criteria

Questionnaires that were completed incompletely or exhibited invalid response patterns (such as identical or random responses) were excluded from the final analysis. Additionally, responses from individuals who were outside the specified age range or reported residing outside of Semnan were disregarded.

### 2.4 Research Instruments

To collect data, three standardized questionnaires were used:

1. Self‐compassion scale (SCS); Neff et al. 2003: This scale consists of 26 items across six components (self‐kindness, self‐judgment, common humanity, isolation, mindfulness, and over‐identification). Responses are given on a 5‐point Likert scale (1 = strongly disagree to 5 = strongly agree), with some items being reverse‐scored. The Persian revised version of the SCS demonstrated a six‐factor structure, with good internal consistency (Cronbach's α = .91) and acceptable convergent validity (r = −0.45 with the General Health Questionnaire) in an Iranian sample (Jelodari and Gheydari 2016).
2. Meaning in Life Questionnaire (MLQ); Steger et al. 2006: This 10‐item scale measures the experience of meaning in life. Responses are given on a 7‐point Likert scale (1 = strongly false to 7 = strongly true), with item 9 being reverse‐scored. A sample item is: [2. I am searching for something that gives meaning to my life]. The Farsi version of the MLQ has demonstrated good psychometric properties in an Iranian sample, with excellent internal consistency (Cronbach's α = 0.96) and test–retest reliability (ICC = 0.98), as well as evidence of content, convergent, and factorial validity (Sharif Nia et al. 2023).
3. Childhood Trauma Questionnaire (CTQ); Bernstein et al. 2003: This scale includes 28 items and five components (emotional, physical, and sexual abuse and emotional and physical neglect). Twenty‐five items are designed to measure the components, and three items are for detecting denial of childhood problems. A sample item is [8.I thought my parents wished I had never been born]. The Persian version of the CTQ demonstrated acceptable reliability (Cronbach's α = 0.79; test–retest reliability = 0.90) and convergent validity (Garrusi and Nakhaee 2009).

### 2.5 Ethical Considerations

The ethical principles of the research included informed consent, voluntary participation, confidentiality, and anonymity of the data. Informed consent was obtained at the beginning of the online questionnaire, and no personally identifiable information was collected. The study received no financial support and was conducted entirely at the researcher's own expense. Given the low‐risk nature of the study, formal institutional ethics approval was not required; however, all procedures were conducted in accordance with the declaration of Helsinki (2013) and with full respect for participants’ consent and confidentiality.

### 2.6 Structural Equation Modeling (SEM)

To examine the relationships between childhood trauma and meaning in life, considering self‐compassion as a mediating variable, Partial Least Squares Structural Equation Modeling (PLS‐SEM) was employed. Data analysis was conducted using SmartPLS software. Prior to conducting the PLS‐SEM analysis, Harman's single‐factor test was performed to assess potential common method bias.

## 3 Results

### 3.1 Demographic Characteristics of the Sample

The research sample consisted of 562 individuals. In terms of gender, women accounted for the largest share with 346 participants (61.6%), while men comprised 216 participants (38.4%). The largest age group was individuals aged 18 to 25, with 272 participants (48.4%), and other middle‐aged groups were also represented with appropriate distribution in the sample. Regarding economic status, the majority of respondents were at an average level, with 333 participants (59.3%), while the representation of economic groups at both extremes (very poor or very good) was lower. This distribution provides a clear picture of the sample composition and offers a suitable context for interpreting the research results.

### 3.2 Description and Examination of the Main Variables of the Research

Table 1 shows the descriptive statistics of the main variables in the research. This table includes the mean, standard deviation, skewness, and kurtosis of each subscale and the total score for the variables of childhood trauma, self‐compassion, and meaning in life, providing a clear picture of the initial status of the constructs in the research sample.

**TABLE 1:** Description and examination of the main variables of the research.

Variable	Subscale	Minimum	Maximum	Mean	Standard Deviation	Skewness	Kurtosis
**Childhood Trauma**	Emotional abuse	5	19	7.66	3.23	1.107	0.935
	Physical abuse	5	21	7.27	3.30	1.920	3.181
	Sexual abuse	5	20	7.47	3.09	1.439	1.677
	Emotional neglect	5	25	11.95	5.20	0.558	−0.712
	Physical neglect	5	21	9.38	3.39	0.845	0.278
	Total Childhood Trauma	25	95	43.76	14.08	0.967	0.481
**Self‐Compassion**	Self‐kindness	10	25	18.32	3.92	−0.292	−0.781
	Self‐judgment	5	23	11.99	3.38	0.280	0.273
	Common humanity	8	20	15.00	2.65	−0.557	0.091
	Isolation	4	20	9.57	3.17	0.539	−0.048
	Mindfulness	8	20	15.02	2.26	−0.246	0.174
	Over‐identification	4	19	9.12	2.97	0.395	0.266
	Total Self‐Compassion	50	120	79.03	12.56	0.016	0.052
**Meaning in Life**	Search for meaning	5	35	29.27	5.58	−1.536	2.775
	Presence of meaning	5	35	26.15	7.62	−0.828	−0.174
	Total Meaning in Life	10	70	55.43	11.48	−0.885	0.692

As shown in Table 1, childhood trauma was found to be at a relatively low level, indicating that the intensity of trauma experiences in the sample was generally low. Self‐compassion was at an average level, reflecting a moderate degree of self‐compassion among participants. In contrast, meaning in life was at a higher level, suggesting that most individuals reported a significant perception of meaning and direction in life. The distribution of scores for all three variables was approximately symmetrical and deemed suitable for statistical analysis.

### 3.3 Examination of the Assumptions of Structural Equation Modeling

Before conducting structural equation modeling, the fundamental assumptions of the analysis, including the normality of the variables and the presence of correlations among the variables, were examined.

According to Table 2, the tests indicated that the distribution of the variables was statistically non‐normal; however, SmartPLS software is not sensitive to the normality of the data and is suitable for model analysis.

**TABLE 2:** Examination of the normality of the research variables using the Kolmogorov‐Smirnov Test.

Variable	K–S statistic	Significance (*p*)
Childhood trauma	0.123	0.001
Self‐compassion	0.076	0.001
Meaning in life	0.102	0.001

Additionally, the examination of correlations showed that childhood trauma had a negative relationship with self‐compassion and meaning in life, while self‐compassion had a positive and significant relationship with meaning in life.

Based on Table 3, these results indicate that an increase in traumatic experiences is associated with a decrease in self‐compassion and perceived meaning, while self‐compassion is related to an increase in feelings of meaning and direction in life.

**TABLE 3:** Examination of the correlation among research variables using the Spearman Correlation Test.

Variable	Childhood trauma	Self‐compassion	Meaning in life
Childhood trauma	1		
Self‐compassion	−0.337^**^	1	
Meaning in life	−0.357^**^	0.441^**^	1

** Significant at the 0.01 level.

### 3.4 Assessment of Common Method Bias

Given that the data were collected using self‐report questionnaires from the same participants, the potential for common method bias was assessed using Harman's single‐factor test. All measurement items were entered simultaneously into an unrotated exploratory factor analysis. The results showed that the first factor accounted for 23% of the total variance, which was below the commonly used 50% threshold. These findings suggest that common method bias is unlikely to have substantially influenced the study's results.

### 3.5 Model Fit of Measurement Models

Before examining the results of structural equation modeling, the reliability and validity of the measurement models and their fit indices were analyzed.

To assess the structural validity of the instruments and the degree of correlation of each item with the specified theoretical factors, confirmatory factor analysis (CFA) was conducted. Table 4 presents the standardized factor loadings obtained from the CFA, indicating the strength of the relationship of each item with its corresponding factor.

**TABLE 4:** Examination of factor loadings (confirmatory factor analysis) for each variable in the model.

Item	Childhood trauma	Item	Self‐compassion	Item	Meaning in life
ct11	0.601	sc10	0.560	m1	0.797
ct12	0.596	sc12	0.671	m10	0.583
ct13	0.813	sc14	0.509	m2	0.596
ct14	0.678	sc15	0.676	m3	0.650
ct15	0.585	sc17	−0.462	m4	0.847
ct16	−0.807	sc18	0.599	m5	0.831
ct17	0.518	sc19	0.725	m6	0.854
ct18	0.723	sc20	0.525	m7	0.747
ct19	0.822	sc21	0.708	m8	0.603
ct2	0.638	sc23	0.671	m9	0.769
ct22	−0.819	sc26	0.678		
ct24	0.457	sc3	0.564		
ct25	0.443	sc5	0.616		
ct26	0.511	sc6	0.723		
ct27	0.471	sc8	0.542		
ct28	0.852	sc9	0.547		
ct3	0.686				
ct4	0.686				
ct5	0.645				
ct7	0.705				
ct8	0.678				
ct9	0.586				

As shown in Table 4, the examination of factor loadings for the remaining items for all three constructs—childhood trauma, self‐compassion, and meaning in life—had acceptable factor loadings, predominantly in the moderate to strong range. Some items with high loadings indicated strong correlations with the latent factor, while items with weak loadings were removed, which helped refine the scales and improve construct validity. Six items related to childhood trauma (1, 6, 10, 20, 21, 23) and ten items related to self‐compassion (2, 4, 7, 10, 11, 13, 16, 22, 24, 25) were eliminated.

To assess the reliability of the research constructs, Cronbach's alpha was first used as a traditional measure, followed by composite reliability as a more modern measure. Composite reliability, introduced by Werts et al. (1974), provides higher accuracy in structural equation modeling, with values above 0.7 indicating adequate internal consistency. Table 5 presents the results of Cronbach's alpha and composite reliability for the research variables.

**TABLE 5:** Examination of Cronbach's alpha and composite reliability for the research variables.

Variable	Cronbach's alpha	Composite reliability (CR)
Childhood trauma	0.876	0.908
Self‐compassion	0.857	0.888
Meaning in life	0.908	0.920

As shown in Table 5, the values of Cronbach's alpha and composite reliability for all constructs were above 0.7, thus confirming the reliability of the constructs and indicating adequate internal consistency among the items of each construct.

Convergent validity was assessed using the average variance extracted (AVE). This index indicates the degree of correlation of the construct with its indicators, and a value above 0.5 is considered sufficient. Table 6 presents the AVE values for the research constructs.

**TABLE 6:** Examination of the average variance extracted (AVE) values.

Variable	Average variance extracted (AVE)
Childhood trauma	0.539
Self‐compassion	0.510
Meaning in life	0.540

As shown in Table 6, the AVE for each construct was above 0.5, thus confirming the convergent validity of the model.

Divergent validity was also assessed to examine the uniqueness of the constructs using the Fornell and Larcker criterion. According to this criterion, the square root of the AVE for each construct should be greater than its correlation with other constructs. The results indicated that the square root of the AVE for each construct was higher than the correlation values among the constructs. Table 7 presents the divergent validity matrix based on the Fornell and Larcker criterion.

**TABLE 7:** Divergent validity matrix using the Fornell and Larcker method.

Variable	Childhood trauma	Self‐compassion	Meaning in life
Childhood trauma	**0.662**		
Self‐compassion	−0.462	**0.616**	
Meaning in life	−0.433	0.557	**0.735**

As shown in Table 7, the constructs have greater interaction with their own indicators, confirming the divergent validity of the model.

### 3.6 Structural Model Fit and Path Analysis

**FIGURE 2:**
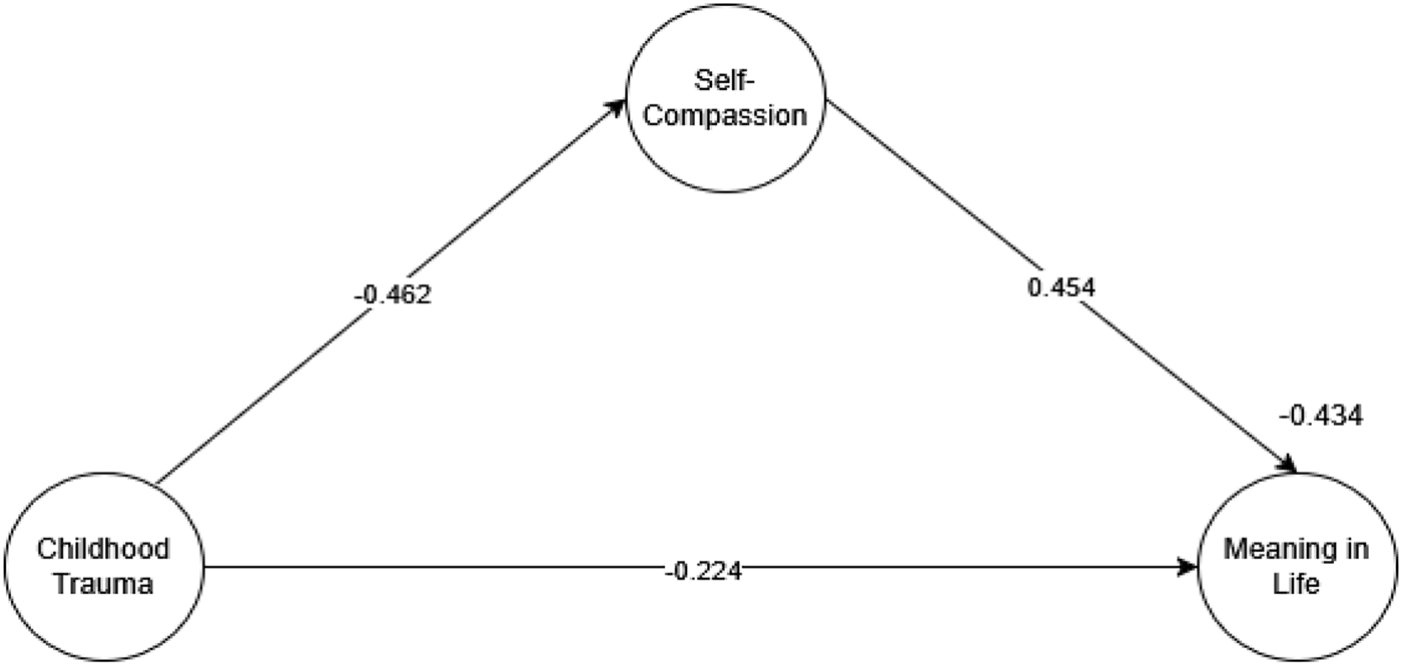
Structural model of the research with standardized path coefficients.

To examine the structural relationships between childhood trauma, self‐compassion, and meaning in life, the conceptual model of the research was tested using structural equation modeling. After confirming the model fit, the path coefficients for direct and indirect relationships among the research variables were estimated. Figure 2 illustrates the structural model with standardized path coefficients.

To investigate the structural relationships between childhood trauma, self‐compassion, and meaning in life, the path coefficients for both direct and indirect effects were estimated. Table 8 presents the standardized direct and indirect path coefficients for the proposed mediation model.

**TABLE 8:** Examination of standardized direct and indirect coefficients for the relationship between childhood trauma and meaning in life with self‐compassion as a mediator.

Path	Standardized path coefficient (β)	t‐value	*p*‐value
Childhood trauma → self‐compassion	−0.462	15.838	0.001
Childhood trauma → meaning in life	−0.224	5.861	0.001
Self‐compassion → meaning in life	0.454	13.308	0.001
Childhood Trauma → self‐compassion → meaning in life	−0.210	10.304	0.001

As shown in Table 8, childhood trauma has a significant negative effect on self‐compassion. Additionally, childhood trauma also showed a significant negative effect on meaning in life. On the other hand, self‐compassion had a positive and significant effect on meaning in life. Furthermore, the indirect effect of childhood trauma on meaning in life through self‐compassion was reported as significant, indicating the mediating role of self‐compassion in this relationship.

## 4 Discussion

The aim of the present study was to examine the mediating pathways from childhood trauma to meaning in life, emphasizing the level and type of self‐compassion. The discussion will address each of the research hypotheses.

### 4.1 Hypothesis 1: The Relationship Between Childhood Trauma and Meaning in Life

The findings of the present study indicated that childhood trauma was negatively associated with meaning in life. This finding aligns with previous research (Arpacıoğlu et al. 2024; Rose et al. 2023; Suh and Chong 2021).

From a physiological perspective, childhood trauma may alter HPA‐axis functioning and stress‐related brain systems, particularly the hippocampus, amygdala, and prefrontal cortex. (Niu et al. 2025; Tomoda et al. 2025). These changes may affect emotion regulation and cognitive flexibility, potentially making it more difficult to develop a coherent and meaningful view of life (Sui and Humphreys 2015; King and Hicks 2021).

In fact, when the neural pathways responsible for self‐reflection and narrative coherence are disrupted, the individual struggles to maintain conceptual continuity between the past and present and to preserve their sense of purpose.

From a cognitive perspective, Beck's theory (1967) provides a suitable framework for understanding this relationship. According to this theory, traumatic experiences alter an individual's fundamental belief system and lead to the formation of negative beliefs about the self, others, and the world. These negative beliefs distort cognitive evaluations of events and hinder the positive reconstruction of experiences (Milman et al. 2020). As a result, mental coherence and a purposeful understanding of life are weakened, leading to a decreased experience of meaning.

Within the framework of Young's schema theory (2003), this finding can also be explained. Trauma activates schemas such as distrust, entitlement, dependency, self‐sacrifice, and harsh standards, disrupting the individual's semantic system at a deep emotional‐cognitive level. These schemas cause individuals to interpret negative events not in terms of growth or meaning but as affirmations of their worthlessness (Orang et al. 2017). Therefore, the individual's ability to integrate painful experiences into a coherent and purposeful framework is diminished, reinforcing feelings of emptiness and meaninglessness (Arpacıoğlu et al. 2024).

In summary, trauma affects meaning in life through four complementary pathways: first, neurobiological changes; second, dysfunctional cognitive beliefs; third, maladaptive early schemas; and fourth, an unstable environment. These four pathways synergistically lead to a decrease in the experience of meaning in life and an increase in existential helplessness. Individuals who have experienced childhood trauma may derive meaning from positive relationships, daily routines, leisure moments, artistic activities, future goals, and sometimes spirituality. However, this experience of meaning is accompanied by mistrust, fear, and psychological harm resulting from trauma (Olstad et al. 2023).

### 4.2 Hypothesis 2: The Relationship Between Trauma and Self‐Compassion

The results of this study indicate that self‐compassion, as a psychological protective factor, can mitigate the negative effects of childhood trauma. This finding is consistent with previous studies (Collins et al. 2023; Zhang et al. 2021).

The observed negative association may reflect the long‐term impact of adverse caregiving experiences on individuals’ internal representations of themselves and others. Experiences of abuse and neglect may foster self‐criticism and reduce the tendency to respond to personal difficulties with kindness and acceptance. From an attachment perspective, these patterns may persist into adulthood and help explain why greater childhood trauma was associated with lower self‐compassion in the present study (Zhang et al. 2021).

However, survivors of childhood trauma who exhibit higher levels of self‐compassion experience lower psychological distress. Self‐compassion enhances mental health and quality of life by reducing negative self‐evaluations and strengthening adaptive coping skills, including self‐soothing and self‐assurance. These skills enable individuals to manage negative reactions to distressing experiences, decrease negative thoughts about themselves, increase self‐kindness, and foster a sense of belonging to the shared human experience (Collins et al. 2023; Tao et al. 2021). Thus, self‐compassion may buffer the psychological consequences of childhood trauma by reducing the impact of self‐criticism on psychopathology, thereby serving as a potential resilience factor among survivors of childhood maltreatment (Lassri and Gewirtz‐Meydan 2022).

### 4.3 Hypothesis 3: The Relationship Between Self‐Compassion and Meaning In Life

The results of this study show that self‐compassion has a positive and significant relationship with meaning in life. This finding is consistent with previous studies (Suh and Chong 2021; Chan et al. 2022; O'Dea et al. 2022).

Self‐compassion allows individuals to engage with negative experiences and failures in a healthy and adaptive manner, facing their pain and difficulties with acceptance and understanding rather than avoidance, blame, or constant criticism. This process not only changes the “content” of negative emotions and thoughts but also reduces self‐critical responses, creating a mental space for healthy self‐regulation and personal growth.

Moreover, self‐compassion enhances intrinsic motivation and reduces reliance on external expectations, enabling individuals to rely more on their personal goals and values, thereby creating more opportunities to experience meaning and life satisfaction (Suh and Chong 2021).

According to Gilbert's social mentality theory, receiving kindness and compassion from others can also strengthen individual self‐compassion; this increases resilience and enables individuals to face life's challenges in a positive and adaptive way. By adopting a kind and non‐judgmental attitude towards themselves, difficult experiences can transform into opportunities for growth and meaning reconstruction, ultimately enhancing feelings of purpose and life satisfaction (Chan et al. 2022). Thus, self‐compassion acts as an effective psychological resource that enhances the sense of meaning; this protective role prevents experiences of boredom, emptiness, and meaninglessness (O'Dea et al. 2022).

### 4.4 Hypothesis 4: The Mediating Role of Self‐Compassion in the Relationship Between Childhood Trauma and Meaning in Life

The findings of the present study indicate that self‐compassion plays a mediating role in the relationship between childhood trauma and meaning in life. Although previous research has identified a protective role for self‐compassion in the association between adverse childhood experiences and meaning in life (Gentry et al. 2025), the present study extends this literature by examining self‐compassion as a mediating pathway between childhood trauma and meaning in life.

This mediation can be explained through several pathways. Individuals with higher levels of self‐compassion respond to negative experiences and past failures with acceptance and kindness towards themselves, experiencing less self‐blame, shame, or constant self‐criticism. This kind attitude towards oneself creates a psychologically safe space for processing negative emotions and traumatic memories, reducing the intensity of anxiety and psychological distress (Collins et al. 2023; Tao et al. 2021).

This interpretation is consistent with attachment theory, as adverse early caregiving experiences may shape enduring patterns of self‐evaluation and interpersonal expectations. It is also consistent with Gilbert's social mentality theory and Neff's conceptualization of self‐compassion, which emphasize the roles of self‐kindness, emotional regulation, and reduced self‐criticism in adaptive responses to adversity. Together, these perspectives suggest that self‐compassion may represent a psychological pathway through which childhood trauma is associated with meaning in life (Chan et al. 2022; Liao et al. 2025).

Through these processes, self‐compassion may help individuals regulate distress, integrate difficult experiences, and maintain personally meaningful goals and values, thereby supporting a stronger sense of meaning and purpose despite childhood adversity (Suh and Chong 2021; O'Dea et al. 2022).

Thus, the mediating effect observed in the present study suggests that self‐compassion may be an important psychological resource for maintaining meaning and purpose among individuals with a history of childhood trauma. However, given the cross‐sectional design, this mediating pathway should be interpreted as an observed statistical association rather than evidence of a causal mechanism.

### 4.5 Limitations

1. A cross‐sectional design with a retrospective self‐report measure of childhood trauma (CT) was used. Therefore, causal relationships cannot be established, and the assessment may be subject to recall bias. In addition, the developmental timing and duration of exposure to CT were not considered.
2. The research sample was selected solely from individuals aged 18–60 residing in urban areas of Semnan; hence, generalizing the results to rural populations and other regions, which may have different experiences, parenting styles, or access to mental health services, should be done cautiously.
3. Due to the use of convenience sampling and voluntary participation, caution should be exercised when generalizing the findings to the broader population.
4. Common Method Bias: Since all variables were assessed using self‐report questionnaires at the same time, common method bias cannot be completely ruled out. Future studies could use multiple data sources or different assessment methods to reduce the potential influence of common method bias

### 4.6 Future Research Suggestions

1. Longitudinal Studies: Utilizing a longitudinal design could clarify the causal relationships among childhood trauma, self‐compassion, and meaning in life, revealing their mediating pathways and long‐term effects.
2. Clinical Samples: Investigating these relationships in clinical populations with psychological disorders, such as depression or anxiety, could demonstrate whether the role of self‐compassion in mitigating the negative outcomes of childhood trauma is similar or different in these groups.
3. Psychological Interventions: Experimental studies could examine the effects of programs and interventions aimed at enhancing self‐compassion to determine how increasing self‐compassion improves the experience of meaning and life satisfaction while reducing the negative consequences of childhood trauma.
4. Additional Mediating and Moderating Variables: Exploring other factors such as social support, resilience, family functioning, identity styles, and individual characteristics could provide a more complex structural model of the impact of trauma on the experience of meaning.

## 5 Conclusion

The findings of the present study indicate that childhood trauma is negatively associated with meaning in life, while self‐compassion is positively associated with meaning in life and mediates this association. These findings suggest that individuals with greater self‐compassion may be better able to respond to adverse experiences with greater self‐acceptance and emotional flexibility, potentially supporting a stronger sense of coherence and purpose in life. The findings highlight self‐compassion as a potentially important psychological resource in understanding the relationship between childhood trauma and meaning in life and suggest that strengthening self‐compassion may be considered in preventive and supportive interventions for individuals affected by childhood adversity.

## Author Contributions

**Reza Refaei**: conceptualization, investigation, funding acquisition, writing – original draft, validation, visualization, writing – review and editing, formal analysis, project administration, supervision, resources, methodology, software, data curation.

## Funding

The author has nothing to report.

## Supporting information




**Supplementary Material**: brb371800‐sup‐0001‐SuppMat.spv

## Data Availability

The datasets used or analyzed during the current study are available from the corresponding author on reasonable request.
